# A python based algorithmic approach to optimize sulfonamide drugs via mathematical modeling

**DOI:** 10.1038/s41598-024-62819-0

**Published:** 2024-05-28

**Authors:** Wakeel Ahmed, Kashif Ali, Shahid Zaman, Fekadu Tesgera Agama

**Affiliations:** 1 Department of Mathematics, University of Sialkot, Sialkot, 51310 Pakistan; 2 COMSATS University Islamabad Lahore Campus, Lahore, Pakistan; 3 Department of Mathematics, Wollega University, 395, Nekemte, Ethiopia

**Keywords:** Sulfonamide Drugs, QSPR Analysis, Linear regression, Topological indices, Python Algorithms, Applied mathematics, Pharmaceutics

## Abstract

This article explores the structural properties of eleven distinct chemical graphs that represent sulfonamide drugs using topological indices by developing python algorithm. To find significant relationships between the topological characteristics of these networks and the characteristics of the associated sulfonamide drugs. We use quantitative structure-property relationship (QSPR) approaches. In order to model and forecast these correlations and provide insights into the structure-activity relationships that are essential for drug design and optimization, linear regression is a vital tool. A thorough framework for comprehending the molecular characteristics and behavior of sulfonamide drugs is provided by the combination of topological indices, graph theory and statistical models which advances the field of pharmaceutical research and development.

## Introduction

Sulfonamide drugs, which contain a sulfonamide functional group, have a significant medical history that dates back to the 1930s when the first synthetic antibacterial agent, Prontosil, was discovered^1^. Since then, they have been widely used for their antibacterial qualities, especially in fighting bacterial infections. In addition to their antibacterial properties, sulfonamide drugs also demonstrate effectiveness against specific protozoal infections, making them highly flexible in the treatment of infectious diseases.Sulfonamide drugs have become a vital class of substances with a wide range of therapeutic uses in the field of pharmaceutical research^2^. Sulfonamide’s drugs are also commonly used for the treatment of urinary tract infections, respiratory tract infections, and bacterial meningitis^3^. They function by limiting the production of folic acid in bacteria, therefore impeding their growth and reproduction. Sulfonamide medications are additionally employed for the treatment of toxoplasmosis and malaria^4^. Sulfonamide’s distinct chemical structure makes them a perfect candidate for optimization in drug development due to their effectiveness against a variety of medical conditions^5^. Customized features of sulfonamide drugs that enhance pharmacological effects require an understanding of their quantitative structure-activity relationship (QSAR)^6,7^. Degree-based Topological Indices are essential for understanding the complex relationships among sulfonamide drugs . These indices, which assign numerical values depending on the connectivity of atoms inside the compound, provide a quantitative representation of the molecular structure^8–11^ . More specifically, a molecule’s topological characteristics are mostly determined by the degree or number of bonds that each atom provides^12,13^.

Degree-based topological indices that are used in this study presented in Table 1 , which show the spatial arrangement and connectivity of atoms, offer significant novel perspectives on the structural characteristics of sulfonamide drugs that affect their biological behavior^14^. The application of these indices is essential in comprehending the complicated relationships between structure and function, particularly with complex molecular structures^15^. This aids researchers in designing and refining Sulfonamide compounds in a rational way in order to optimize their pharmacological effects^16^. A QSPR analysis is based on the correlation between these indices and the biological activity of sulfonamide drugs. Utilizing mathematical techniques like linear regression makes it possible to systematically examine the structure-activity landscape and identify patterns that inform the optimization of potential sulfonamide drugs candidates. Several researchers have recently made contributions in this domain^17–20^.

A Python program has been developed with the goal of obtaining a thorough understanding and practical application of these relationships. This application streamlines the QSPR analysis process by facilitating the application of mathematical models and the computation of topological indices. Scientists and researchers may quickly optimize sulfonamide medications, find hidden relationships and analyze massive data sets efficiently by incorporating the Python application into their workflow. This integrated strategy which combines Python programming, degree-based topological indices, QSPR analysis and sulfonamide drug research, advances pharmaceutical development and advances the continuous seek for novel and more effective therapeutic agents.

**Table 1:** Different topological descriptors.

Gutman and Polansky^21^	$M_{1}(G)=\sum _{v w \in E(G)}\left( d_{v}+d_{w}\right)$	First Zagreb index
	$M_{2}(G)=\sum _{v w \in E(G)}\left( d_{v} \times d_{w}\right)$	Second Zagreb index
Fatjlowicz^22^	$H(G)=\sum _{v w \in E(G)} \frac{2}{\left( d_{v}+d_{w}\right) }$,	Harmonic index
Furtula and Gutman^23^	$F(G)=\sum _{v w \in E(G)}\left( d_{v}\right) ^{2}+\left( d_{w}\right) ^{2}$,	Forgotten index
Zhao^24^	$S S(G)=\sum _{v w \in E(G)} \sqrt{\frac{d_{v} \times d_{w}}{d_{v}+d_{w}}}$	SS index
Ranjini^25^	${\text {ReZG}}_{2}(G)=\sum _{v w \in E(G)} \frac{d_{v} \times d_{w}}{d_{v}+d_{w}}$	Re-define second Zagreb index
	${\text {ReZG}}_{3}(G)=\sum _{v w \in E(G)}\left( d_{v} \times d_{w}\right) \left( d_{v}+d_{w}\right)$	third Zagreb index

## Methodology

We firstly convert chemical structures into molecular graphs and edge partitioning is performed, based on graph connectivity. Secondly, Degree-based topological indices were computed by analyzing the distribution of node degrees within the graph by developing python algorithm. For python program we import necessary library numpy then define different variables for edge-partition and lastly apply for-loop to compute indices. Furthermore we use SPSS software for Regression analysis to assess the connection between the computed indices and experimental characteristics. To evaluate the developed indices ability to predict molecular behavior, a comparison of actual and predicted values was made.

## Results and discussion

**Figure 1:**
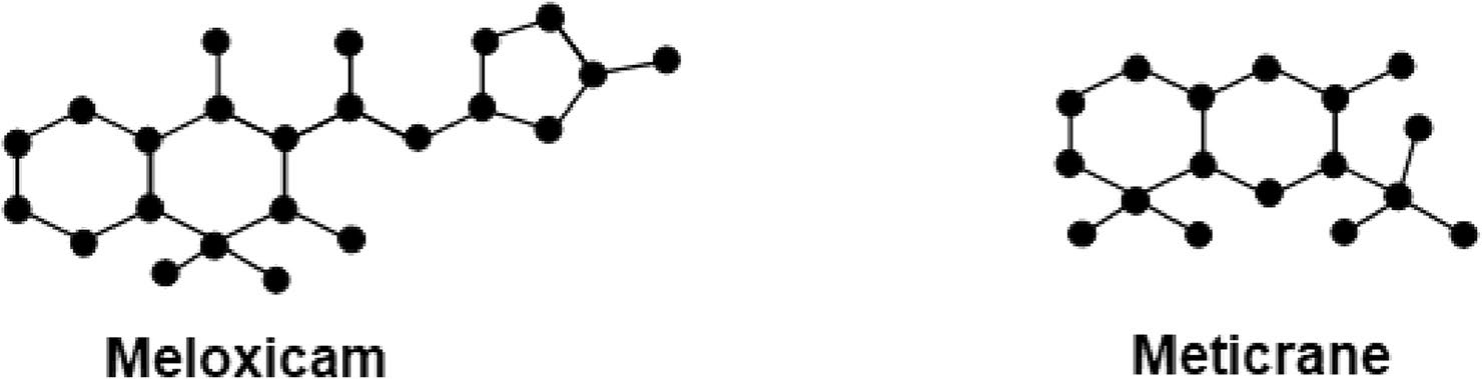
Molecular Graph of Meloxicam and Meticrane.

**Figure 2:**
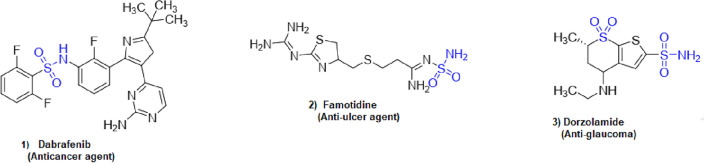
Molecular structure of Dabrafenib,Famotidine and Dorzolamide.

**Figure 3:**
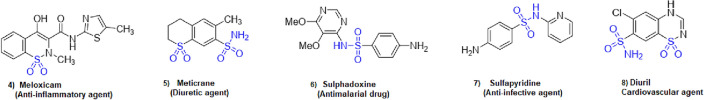
Molecular structure of Sulfonamide Drugs.

**Figure 4:**
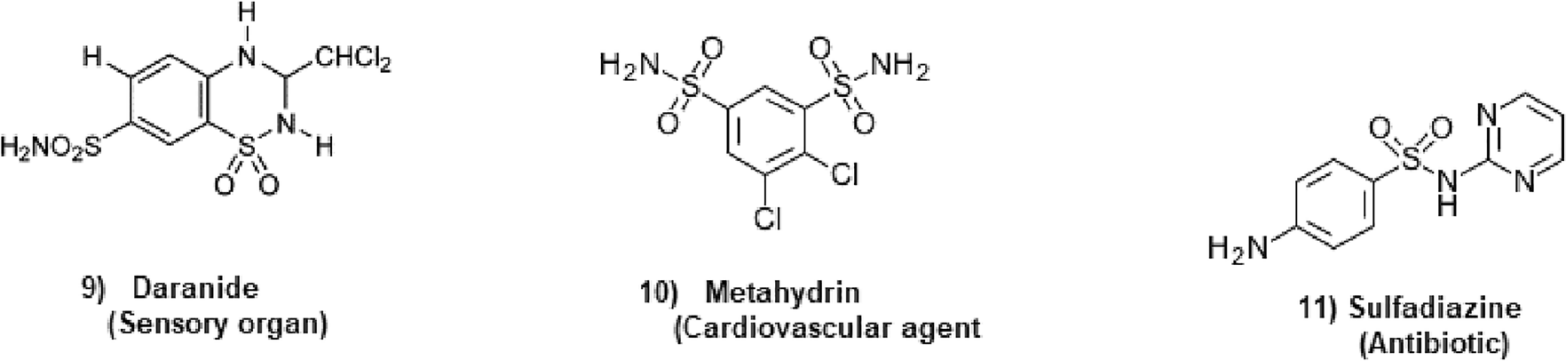
Molecular structure of Daranide, Metahydrin and Sulfadiazine.


Chemical graphs representing the molecular structures of sulfonamide drugs shown in Fig. 1 were used to start the QSPR analysis. A systematic representation of the complex connection patterns within each molecule was made possible by this change. The topological indices of these chemical graphs were determined by developing an edge-partitioning-based Python Algorithms. The degree-based topological characteristics that are essential for comprehending the structural subtleties affecting the pharmacological characteristics of sulfonamide drugs were successfully captured by this approach. Linear regression analysis was carried out using the Statistical Package for the Social Sciences (SPSS) to uncover the statistical correlations between the biological activity of the sulfonamide compounds and the computed topological indices. By identifying important links, this stage helped to clarify the essential topological aspects that underlie the biological effects that have been observed. Also, a Python algorithm is developed especially for the comparison section to guarantee the analysis’s resilience and dependability. This approach made it possible to thoroughly analyze and validate the linear regression findings, offering a rigorous assessment of the topological indices’ predictive power in clarifying the structure-activity relationship of sulfonamide drugs. polymers of sulfonamides. The topological indices for a group of sulfonamide drugs shown in Figs. 2, 3 and 4 have been determined using Algorithm-1 and Algorithm-2 presented in Table 2.

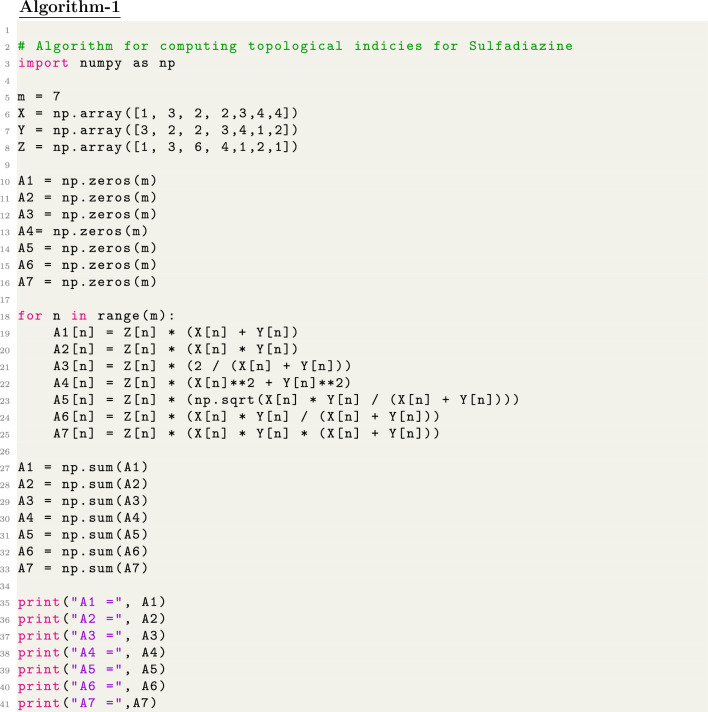



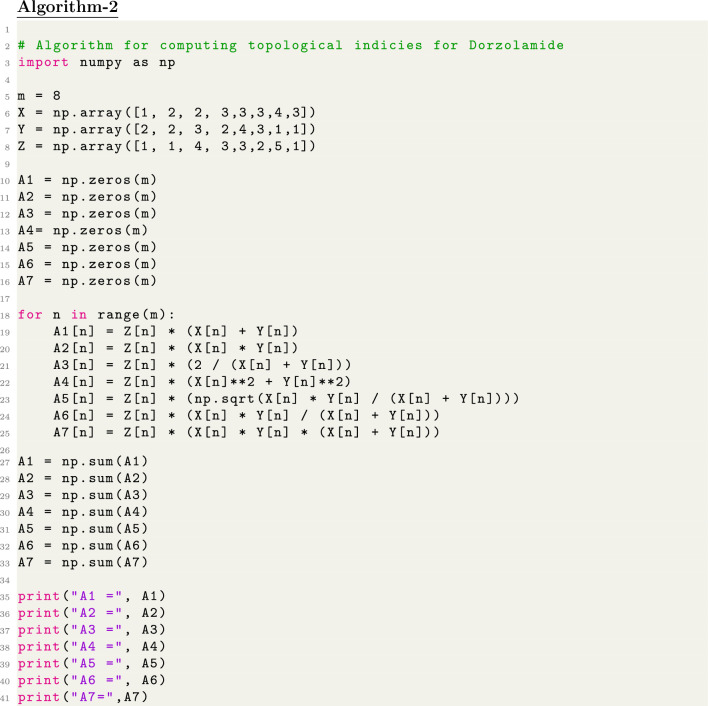


## Regression model

A linear equation in the form of $Y = A + BX$ demonstrates the relationship between the independent variables (X) and the dependent variable (Y) in linear regression. In this case, Y is the dependent variable’s predicted or estimated value, X is the independent variable, ’B’ denotes the regression line’s slope, and ’A’ is the y-intercept. ’B’ and ’A’ values that minimize the difference between the expected and actual observed values are the ones that need to be found. As linear regression models enable researchers to investigate and measure the relationships between different molecular parameters and the possible efficacy of treatment candidates, therefore linear regression models are crucial resources for molecular insights into anti-Alzheimer’s medications. Below we have computed sevral linear regression models with respect to TIs discussed in Table 2.

Regression models of $M_1(G)$

- Polarizability = 16.5920 + 0.1177 $$M_1(G)$$
- Complexity = 253.1917 + 2.1931 $$M_1(G)$$
- Boiling point = 562.5737 + 0.0892 $$M_1(G)$$
- Molecular weight = 205.7471 + 1.0540 $$M_1(G)$$
- Molecular volume = 110.2459 + 0.8195 $$M_1(G)$$
- Flash point = 294.0339 + 0.0539 $$M_1(G)$$

Regression models of $M_2(G)$

- Polarizability = 7.5184 + 0.1832 $$M_2(G)$$
- Complexity = 125.5086 + 3.0798 $$M_2(G)$$
- Boiling point = 510.5981 + 0.5039 $$M_2(G)$$
- Molecular weight = 113.6467 + 1.7289 $$M_2(G)$$
- Molecular volume = 31.4493 + 1.4024 $$M_2(G)$$
- Flash point = 262.5657 + 0.3051 $$M_2(G)$$

Regression models of *H*(*G*)

- Polarizability = 5.0573 + 2.9345 *H*(*G*)
- Complexity = 125.4552 + 44.4959 *H*(*G*)
- Boiling point = 496.9339 + 8.8771 *H*(*G*)
- Molecular weight = 101.2243 + 26.4279 *H*(*G*)
- Molecular volume = 24.4009 + 21.0829 *H*(*G*)
- Flash point = 254.2942 + 5.3739 *H*(*G*)

Regression Models of *F*(*G*)

- Polarizability = 5.6774 + 0.0798 *F*(*G*)
- Complexity = 85.4367 + 1.3719 *F*(*G*)
- Boiling point = 491.9821 + 0.2638 *F*(*G*)
- Molecular weight = 88.2033 + 0.7798 *F*(*G*)
- Molecular volume = 14.0497 + 0.6219 *F*(*G*)
- Flash point = 251.3014 + 0.1597 *F*(*G*)

Regression models of *SS*(*G*)

- Polarizability = 5.67009 + 1.1078 *SS*(*G*)
- Complexity = 115.7992 + 3.2629 *SS*(*G*)
- Boiling point = 500.7366 + 3.2629 *SS*(*G*)
- Molecular weight = 101.4689 + 10.2152 *SS*(*G*)
- Molecular volume = 23.5397 + 8.1969 *SS*(*G*)
- Flash point = 256.5959 + 1.9753 *SS*(*G*)

Regression models of $Rez G_2(G)$

- Polarizability = 6.6479 + 0.9945 $$Rez G_2(G)$$
- Complexity = 127.9736 + 15.9940 $$Rez G_2(G)$$
- Boiling point = 507.9909 + 2.7443 $$Rez G_2(G)$$
- Molecular weight = 110.4326 + 9.1732 $$Rez G_2(G)$$
- Molecular volume = 29.1663 + 7.4271 $$Rez G_2(G)$$
- Flash point = 260.9856 + 1.6614 $$Rez G_2(G)$$

Regression models of $Rez G_3(G)$

- Polarizability = 9.0849 + 0.0316 $$Rez G_3(G)$$
- Complexity = 138.6683 + 0.5511 $$Rez G_3(G)$$
- Boiling point = 517.5597 + 0.0829 $$Rez G_3(G)$$
- Molecular weight=124.7707 + 0.3038 $$Rez G_3(G)$$
- Molecular volume = 39.1387 + 0.2484 $$Rez G_3(G)$$
- Flash point = 266.7802 + 0.0502 $$Rez G_3(G)$$

**Table 2:** The molecular descriptors for the candidate drugs.

Name of drug	$M_1(G)$	$M_2(G)$	*H*(*G*)	*F*(*G*)	*SS*(*G*)	$Rez G_2(G)$	$Rez G_3(G)$
Sulfadiazine	86	97	7.719	228	18.787	19.7976	490
Dorzolamide	104	125	7.9905	310	21.2002	22.9595	704
Meloxicam	226	153	10.2381	350	26.7589	29.1286	830
Sulphadoxine	107	128	7.8714	349	21.4299	23.3286	736
Meticrane	94	111	7.0714	282	19.0382	20.5119	618
Famotidine	94	99	8.6333	250	20.2951	20.7833	478
Dabrafenib	192	229	15.4381	538	40.5236	43.8619	1228
Diuril	91	102	7.2857	267	18.6588	19.631	544
Daranide	84	96	6.2381	260	16.5485	17.5286	540
Metahydrin	104	122	7.8714	310	21.0951	22.6619	674
Sulfapyridine	86	97	7.7190	228	18.787	19.7976	490

**Table 3:** The properties of drugs related to their Physico-chemical characteristics.

Name of drug	Polarizability $cm^3$	Complexity	$B.P^oC$ (760 mmHg)	Molecular weight	Molecular volume	Flash point
Sulfadiazine	25	327	512.6	250.28	167.3	263.8
Dorzolamide	29.9	534	575.8	324.4	211	302
Meloxicam	34.1	628	520.9	351.4	219.6	268.8
Sulphadoxine	30.1	420	522.8	310.33	215.3	270
Meticrane	25.6	485	549.1	275.3	188.1	285.9
Famotidine	31.3	469	662.4	337.5	183.6	354.4
Dabrafenib	50.5	817	653.7	519.6	359.9	349.2
Diuril	24.5	532	608.8	295.7	144	322
Daranide	24.3	452	590.5	305.2	171.2	310.9
Metahydrin	30.5	571	631.3	380.7	217.7	335.6
Sulfapyridine	25.9	331	473.5	249.29	174.1	240.2

The physico-chemical properties listed in Table 3 serve as essential descriptors for the desired molecular properties. The development of QSPR model requires these characteristics. In this case, evaluating the dependability and predictive capability of the QSPR model depends significantly on statistical measures like the correlation coefficient (r), standard error (S.E. ), F-statistic, and p-value. Tables 4, 5, 6, 7, 8, 9, 10, and 11 provide an overview of these statistical measures that shed light on the strength and importance of the correlations between the topological indices and the reported physico-chemical properties. These statistical parameters guarantee a thorough assessment of the model’s performance, allowing scientists to determine how well the model predicts the desired molecular attributes using the topological indices that are specified.

**Table 4:** Correlation coefficients of T.I with respect to different physical characteristics.

T.I	Polarizability	Complexity	Boiling point	Molecular weight	Molar Volume	Flash Point
$M_1(G)$	0.7466	0.7532	0.6811	0.6644	0.6843	0.681
$M_2(G)$	0.9587	0.8722	0.3173	0.8986	0.9656	0.3176
H(G)	0.9745	0.7997	0.3547	0.8718	0.9213	0.3551
F(G)	0.9350	0.8695	0.3718	0.9071	0.9584	0.37201
SS(G)	0.9803	0.8456	0.3475	0.8980	0.9546	0.3478
$RezG_2(G)$	0.9756	0.8492	0.3239	0.8939	0.9588	0.3243
$RezG_3(G)$	0.9228	0.8707	0.2914	0.8809	0.9542	0.2917

**Table 5:** The statistical parameters employed in the QSPR model with respect to $M_1(G)$.

	A	B	r	$r^2$	S.E	F	P
Polarizability	16.5920	0.1177	0.7466	0.5574	5.2603	11.3342	0.008
Complexity	253.1917	2.1931	0.7532	0.5673	96.0935	11.8013	0.007
B.P	562.5737	0.0892	0.06811	0.0046	65.5516	0.0419	0.842
M.W	205.7471	1.0540	0.6644	0.4414	59.4897	7.1119	0.025
M.V	110.2459	0.8195	0.6843	0.4682	43.8155	7.9250	0.020
F.P	294.0339	0.0539	0.0681	0.0046	39.6459	0.0419	0.842

**Figure 5:**
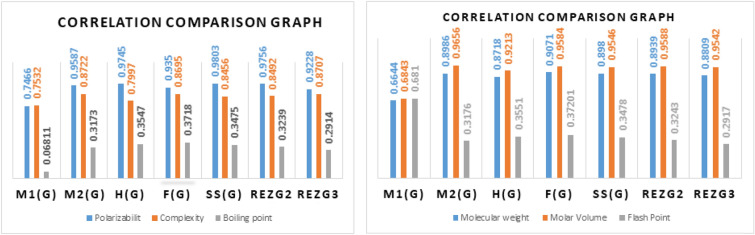
Correlation coefficients with respect to TIs disscused in Table 2.

The correlation coefficients between particular topological descriptors and physico-chemical parameters are shown in Table 4. Interestingly, Polarizability has a significant linear relationship with the SS(G) index, as demonstrated by its high coefficient of 0.9803. The $M_2(G)$ index, which measures complexity, shows a strong association with a coefficient of 0.8722. Furthermore, Boiling point (B.P) has a 0.6811 correlation coefficient and significantly aligns with the $M_1(G)$ index. The $RezG_3$ index and molecular weight (M.W) have a strong association (coefficient of 0.8809), highlighting the topological descriptor’s predictive ability. Furthermore, a good correlation between Molar Volume (M.V) and the $RezG_2$ index is indicated by a high coefficient of 0.9588, indicating a dependable link between the two variables. In Table 5, we have shown the statistical parameters employed in the QSPR model with respect to $M_2(G)$. color redIn Fig. 5, we have shown the correlation coefficients with respect to TIs.

**Table 6:** The statistical parameters employed in the QSPR model with respect to $M_2(G)$.

	A	B	r	$r^2$	S.E	F	P
Polarizability	7.5184	0.1832	0.9587	0.9191	2.2492	102.2225	0.000
Complexity	125.5086	3.07977	0.8722	0.7607	71.4645	28.6095	0.000
B.P	510.5981	0.5039	0.3173	0.1007	62.3089	1.0076	0.341
M.W	113.6467	1.7289	0.8986	0.8075	34.9190	37.7636	0.000
M.V	31.4493	1.4024	0.9656	0.9324	15.6204	124.1682	0.000
F.P	262.5657	0.3051	0.3176	0.1009	37.6808	1.0097	0.341

**Table 7:** The statistical parameters employed in the QSPR model with respect to *H*(*G*).

	A	B	r	$r^2$	S.E	F	P
Polarizability	5.0573	2.9345	0.9745	0.9496	1.7748	169.6371	0.000
Complexity	125.4552	44.4959	0.7997	0.6396	87.7064	15.9698	0.003
B.P	496.9339	8.8771	0.3547	0.1258	61.4309	1.29568	0.284
M.W	101.2243	26.4279	0.8718	0.7600	38.9939	28.5007	0.000
M.V	24.4009	21.0829	0.9213	0.8488	23.3656	50.5158	0.000
F.P	254.2942	5.3739	0.3551	0.1261	37.1488	1.2985	0.283

**Table 8:** The statistical parameters employed in the QSPR model with respect to *F*(*G*).

	A	B	r	$r^2$	S.E	F	P
Polarizability	5.6774	0.0798	0.9350	0.8743	2.8037	62.5779	0.000
Complexity	85.4367	1.3719	0.8695	0.7561	72.1533	27.8948	0.000
B.P	491.9821	0.2638	0.3718	0.1382	60.9948	1.4434	0.260
M.W	88.2033	0.7798	0.9071	0.8228	33.5072	41.7875	0.000
M.V	14.0497	0.6219	0.9584	0.9186	17.1481	101.4976	0.000
F.P	251.3014	0.1597	0.37201	0.1384	36.8851	1.446	0.259

**Table 9:** The statistical parameters employed in the QSPR model with respect to *SS*(*G*).

	A	B	r	$r^2$	S.E	F	P
Polarizability	5.67009	1.1078	0.9803	0.9611	1.5600	222.2048	0.000
Complexity	115.7992	17.6545	0.8456	0.7150	77.9856	22.5826	0.001
B.P	500.7366	3.2629	0.3475	0.1207	61.6097	1.2359	0.295
M.W	101.4689	10.2152	0.8980	0.8064	35.0221	37.4887	0.000
M.V	23.5397	8.1969	0.9546	0.9112	17.9057	92.3453	0.000
F.P	256.5959	1.9753	0.3478	0.1209	37.2571	1.2387	0.294

**Table 10:** The statistical parameters employed in the QSPR model with respect to $RezG_2(G)$.

	A	B	r	$r^2$	S.E	F	P
Polarizability	6.6479	0.9945	0.9756	0.9518	1.7352	177.8857	0.000
Complexity	127.9736	15.9940	0.8492	0.7211	77.1488	77.1488	0.000
B.P	507.9909	2.7443	0.3239	0.1049	62.1605	1.0554	0.331
M.W	110.4326	9.1732	0.8939	0.7991	35.6809	35.7879	0.000
M.V	29.1663	7.4271	0.9588	0.9192	17.0778	102.4097	0.000
F.P	260.9856	1.6614	0.3243	0.1052	37.5907	1.0577	0.330

**Table 11:** The statistical parameters employed in the QSPR model with respect to $RezG_3(G)$.

	A	B	r	$r^2$	S.E	F	P
Polarizability	9.0849	0.0316	0.9228	0.8518	3.0467	51.6164	0.000
Complexity	138.6683	0.5511	0.8707	0.7582	71.8395	28.2179	0.000
B.P	517.5597	0.0829	0.2914	0.0849	62.8520	0.8353	0.384
M.W	124.7707	0.3038	0.8809	0.7759	37.6748	31.1726	0.000
M.V	39.1387	0.2484	0.9542	0.9106	17.9687	91.6356	0.000
F.P	266.7802	0.0502	0.2917	0.0851	38.0099	0.8371	0.384

**Figure 6:**
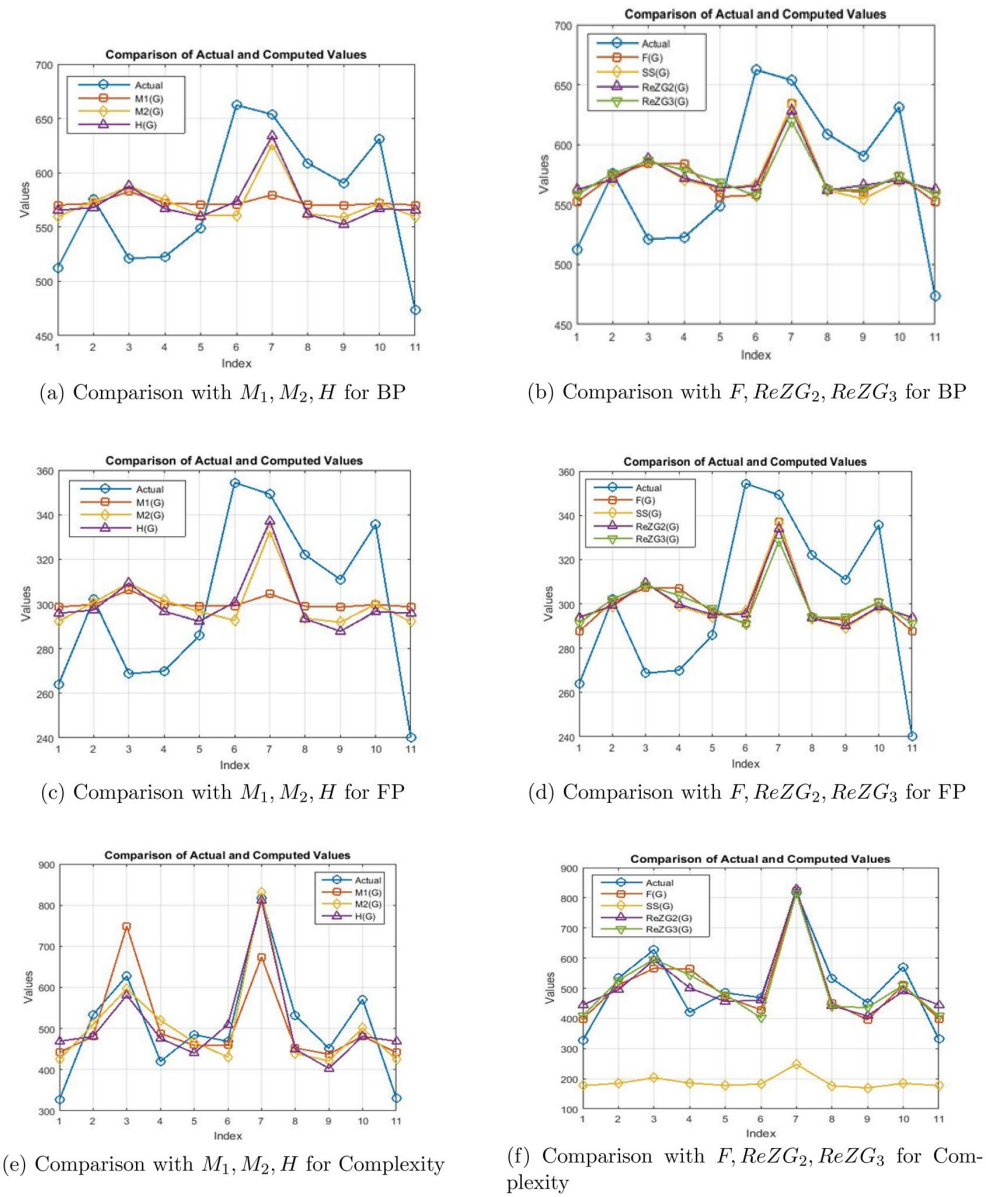
Graphical comparison with actual and predicted values.

Tables 12, 13, 14, 15, 16 and 17 show the computed values of boiling point , flash point, molar volume, molecular weight, complexity, and polarizability that were compared to their corresponding actual values in order to assess the effectiveness of regression models for predicting different physicochemical properties of sulfonamide drugs. In addition to providing insights into the models’ potential utility in forecasting the physicochemical features of sulfonamide drugs and advancing drug development and study, this thorough evaluation is an essential step in demonstrating the models’ robustness and reliability. Also graphical comparison shown in Fig. 6.

**Table 12:** Comparison of actual and computed values of Polarizability from regression models of TIs.

	Polar	$M_1(G)$	$M_2(G)$	H(G)	F(G)	SS(G)	$RezG_2(G)$	$RezG_3(G)$
Sulfadiazine	25	26.7142	25.2888	27.7087	23.8718	26.4823	26.3366	24.5689
Dorzolamide	29.9	28.8328	30.4184	28.5054	30.4154	29.1557	29.4811	31.3313
Meloxicam	34.1	43.1922	35.5480	35.1010	33.6074	35.3136	35.6163	35.3129
Sulphadoxine	30.1	29.1859	30.9680	28.1559	33.5276	29.4101	29.8482	32.3425
Meticrane	25.6	27.6558	27.8536	25.8083	28.1810	26.7606	27.0470	28.6137
Famotidine	31.3	27.6558	25.6552	30.3917	25.6274	28.1530	27.3169	24.1897
Debrafenib	50.5	39.1904	49.4712	50.3604	48.6098	50.5621	50.2686	47.8897
Diurill	24.5	27.3027	26.2048	26.4372	26.9840	26.3403	26.1709	26.2753
Daranide	24.3	26.4788	25.1056	23.3630	26.4254	24.0025	24.0801	26.1489
Metahydrine	30.5	28.8328	29.8688	78.1559	30.4154	29.0392	29.1852	30.3833
Sulfapyridine	25.9	26.7142	25.2888	27.7087	23.8718	26.4823	26.3366	24.5689

**Table 13:** Comparison of actual and computed values of Complexity from regression models of TIs.

	Complex	$M_1(G)$	$M_2(G)$	H(G)	F(G)	SS(G)	$RezG_2(G)$	$RezG_3(G)$
Sulfadiazine	327	441.7983	424.2492	468.9191	398.2299	177.0993	444.6164	408.7073
Dorzolamide	534	481.2741	510.4836	480.9997	510.7257	184.9733	495.1878	526.6427
Meloxicam	628	748.8323	596.7180	581.0087	565.6017	203.1108	593.8564	596.081
Sulphadoxine	420	487.8534	519.7230	475.7002	564.2298	185.7228	501.0912	544.273
Meticrane	485	459.3431	467.3664	440.1035	472.3125	177.9189	456.0409	479.248
Famotidine	469	459.3431	430.4088	509.6017	428.4117	182.0201	460.3817	402.094
Debrafenib	817	674.2669	830.7828	812.3874	823.5189	248.0237	829.5008	815.419
Diurill	532	452.7638	439.6482	449.6390	451.7340	176.6810	441.9518	438.466
Daranide	452	437.4121	421.1694	403.0251	395.4861	169.7453	408.3260	436.2623
Metahydrine	571	481.2741	501.2442	480.9997	510.7257	184.6304	490.4280	510.1097
Sulfapyridine	331	441.7983	424.2492	468.9191	398.2299	177.0993	444.6169	408.707

**Table 14:** Comparison of actual and computed values of Boiling Point from regression models of TIs.

	B.P	$M_1(G)$	$M_2(G)$	H(G)	F(G)	SS(G)	$RezG_2(G)$	$RezG_3(G)$
Sulfadiazine	512.6	570.2449	559.4764	565.4562	552.1285	562.0367	562.3215	558.1807
Dorzolamide	575.8	571.8505	573.5856	567.8664	573.7601	569.9107	570.9987	575.9213
Meloxicam	520.9	582.7329	587.6948	587.8185	584.3121	588.0482	587.9285	586.3667
Sulphadoxine	522.8	572.1181	575.0973	566.8091	584.0483	570.6602	572.0116	578.5741
Meticrane	549.1	570.9585	560.5310	559.7074	556.3737	562.8563	564.2817	568.7919
Famotidine	662.4	570.9585	560.4842	573.5726	557.9321	566.9575	565.0265	557.1859
Debrafenib	653.7	579.7001	625.9912	633.9795	633.9065	632.9611	628.3611	619.3609
Diurill	608.8	570.6909	561.9959	561.6098	562.4167	561.6184	561.8643	562.6573
Daranide	590.5	570.0665	558.9725	552.3101	560.5701	554.7327	566.0946	562.3257
Metahydrine	631.3	571.8505	572.0739	566.8091	573.7601	569.5678	570.1820	573.4343
Sulfapyridine	473.5	570.2449	559.4764	565.4562	552.1285	562.0367	562.3215	558.1807

**Table 15:** Comparison of actual and computed values of Molecular Weight from regression models of TIs.

	M.W	$M_1(G)$	$M_2(G)$	H(G)	F(G)	SS(G)	$RezG_2(G)$	$RezG_3(G)$
Sulfadiazine	250.28	296.3911	281.3500	305.2213	265.9977	293.3819	292.0399	273.6327
Dorzolamide	324.4	315.3621	329.7592	312.3964	329.9413	318.0332	321.0447	338.6459
Meloxicam	351.4	443.9511	378.1684	371.7958	361.1333	374.8164	377.6351	376.9247
Sulphadoxine	310.33	318.5251	334.9459	309.2489	360.3535	320.3796	324.4305	348.3675
Meticrane	275.3	304.8231	305.5546	288.1066	308.1069	295.9479	298.5924	312.5191
Famotidine	337.5	304.8231	284.8078	329.3843	283.1533	308.7874	301.0820	269.9871
Debrafenib	519.6	408.1151	509.5648	509.2209	507.7357	515.4256	482.1637	497.8371
Diurill	295.7	301.6611	289.9945	293.7701	296.4099	292.0723	281.5935	290.0379
Daranide	305.2	294.2831	279.6211	266.0842	290.9513	270.5151	271.2260	288.8227
Metahydrine	380.7	315.3631	324.5725	309.2489	329.9413	316.9596	318.3147	329.5319
Sulfapyridine	249.29	296.3911	281.3500	305.2213	265.9977	293.3819	292.0399	273.6327

**Table 16:** Comparison of actual and computed values of Molecular Volume from regression models of TIs.

	M.V	$M_1(G)$	$M_2(G)$	H(G)	F(G)	SS(G)	$RezG_2(G)$	$RezG_3(G)$
Sulfadiazine	167.3	180.7229	167.4821	187.1398	155.8429	177.5349	176.2051	160.8547
Dorzolamide	211	195.4739	206.7493	192.8638	206.8387	197.3156	199.6888	214.0123
Meloxicam	219.6	295.4529	246.0165	240.2497	231.7147	242.8797	245.5073	245.3107
Sulphadoxine	215.3	197.9324	210.9565	190.3528	231.0928	199.1984	202.4301	221.9611
Meticrane	188.1	187.2789	187.1157	173.4865	189.4255	179.5939	181.5102	192.6494
Famotidine	183.6	187.2789	170.2869	206.4159	169.5247	189.8966	183.5259	157.8739
Debrafenib	359.9	267.5899	352.5989	349.8808	348.6319	355.7076	354.9330	344.1739
Diurill	144	184.8204	174.4941	178.0046	180.0970	176.4840	174.9677	174.2683
Daranide	171.2	179.0839	166.0797	155.9181	175.7437	159.1861	159.3530	173.2747
Metahydrine	217.7	195.4739	202.5421	190.3528	206.8387	196.4541	197.4785	206.5603
Sulfapyridine	174.1	180.7229	167.4821	187.1398	155.8429	177.5349	176.2051	160.8547

**Table 17:** Comparison of actual and computed values of Flash Point from regression models of TIs.

	F.P	$M_1(G)$	$M_2(G)$	H(G)	F(G)	SS(G)	$RezG_2(G)$	$RezG_3(G)$
Sulfadiazine	263.8	298.6693	292.1604	295.7753	287.7130	293.7059	293.8773	291.3782
Dorzolamide	302	299.6395	300.7032	297.2343	300.8084	298.4727	299.1305	302.1210
Meloxicam	268.8	306.2153	309.2460	309.3127	307.1964	309.4528	309.3799	308.4462
Sulphadoxine	270	299.8012	301.6185	296.5943	307.0367	298.9264	299.7437	303.7274
Meticrane	285.9	299.1005	296.4318	292.2952	296.3368	294.2021	295.0641	297.8038
Famotidine	354.4	299.1005	292.7706	300.6887	291.2264	296.6848	295.5150	290.7758
Debrafenib	349.2	304.3827	332.4336	337.2570	337.2200	336.6422	333.8578	328.4258
Diurill	322	298.9388	293.6859	293.4468	293.9413	293.4526	293.6005	294.0890
Daranide	310.9	298.5615	291.8553	287.8171	292.8234	289.2842	290.1076	293.8882
Metahydrine	335.6	299.6395	299.7879	296.5943	300.8084	298.2651	298.6361	300.6150
Sulfapyridine	240.2	298.6693	292.1604	295.7753	287.7130	293.7059	293.8773	291.3782

## Conclusion

A Python algorithm is developed to compute degree-based topological indices, which were then used to examine eleven different sulfonamide drugs. This approach has yielded important insights into the chemical features of these drugs. After that, a regression model isused to determine the characteristics of these drugs, and the results showed that Polarizability, Complexity, Molecular Weight, and Molar Volume were significant factors. These results imply that the behavior and characteristics of sulfonamide drugs are substantially influenced by these particular molecular properties. Unexpectedly, the analysis also indicates that the regression model determined that Boiling Point and Flash Point were not significant indicators. This suggests that both of these factors may have a limited impact on the observed variances in the sulfonamide drugs under consideration within the framework of this research. Our study improves research processes’ transparency and reproducibility by employing a Python software. Since the software code is publicly available, other researchers can independently validate our findings and repeat our methods. This openness encourages scientific integrity and makes it easier for researchers to work together.

## Data Availability

All data generated or analysed during this study are included in this published article.
